# A military suicide prevention program in the Israeli Defense Force: a review of an important military medical procedure

**DOI:** 10.1186/s40696-015-0007-y

**Published:** 2015-09-02

**Authors:** Leah Shelef, Lucian Laur, Gil Raviv, Eyal Fruchter

**Affiliations:** 1 Israeli Air Force, Psychological Branch, Ramat Gan, Israel; 2 Mental Health Unit, Israeli Defense Force Medical Corps, Ramat Gan, Israel; 3 Directorate of Mental Health, Meuhedet Health Maintenance Organization (HMO), Tel Aviv, Israel

**Keywords:** Suicide prevention programs, Suicidality, Military suicide, Adolescents, Israeli soldiers

## Abstract

The phenomenon of suicide during military service is not unique to the Israeli military and other armies. Soldiers’ age––adolescence––is a known factor contributing to suicide, in light of psychological processes of identity formation and self-definition, the stresses of military service, and above all, the availability of weapons. The stigma of seeking help deters some soldiers from getting the assistance they need when they need it most, thus contributing to the higher suicide rate. In the previous decade the IDF initiated intensive and structured preventive procedures aimed at reducing suicide rate among soldiers. The IDF’s Suicide Prevention Program (SPP) was grounded in professional knowledge and backed by military policy changes, both critical to the implementation and change processes. The SPP includes thorough psycho-education and guidance, supervision, greater accessibility of mental health officers, and lower accessibility of nonessential weapons. The SPP has succeeded in reducing the suicide rate by almost 50 %.

The aim of this article is to review the background of the design of the IDF’s SPP and its major components, leading to the current success.

## Introduction

The phenomenon of suicide during military service is not unique to the Israeli Defense Force (IDF). It is a global occurrence, investigated by researchers of various armies [1–7]. There are several known elements contributing to the elevation in suicide rate. One of the most critical elements is the high accessibility to weapons [5, 8–10].

Another group of contributing factors is related to adolescent characteristics: emotional turmoil, identity formation, and self- identity processes [11–13]. Other factors relate to military service stressors [12–14] and to the stigma of seeking assistance within the army [14–16].

The reasons for suicide within the IDF are similar to those found in foreign armies among adolescent soldiers [17].

Military service in Israel is compulsory and perceived as a normative stage of the maturation process for every young male and female, with the majority of adolescents aged 18–21 being drafted into service [13]. Males serve for three years, while females serve for two years [12]. The main justification for the development of a suicide prevention program relates to the fact that, since IDF service is compulsory, with eligible soldiers being obligated to serve by law, therefore, the army assumes legal and moral responsibility for the soldiers’ physical and mental health. Also relevant is the understanding that conscripted soldiers represent a mentally healthy population [12], as they undergo a series of pre-conscription tests and examinations in order to determine their suitability for service [13]. Likewise, with the IDF’s being a small army in a small country, the enormous human tragedy generated by the loss of each soldier also carries significant impact.

## Review

### Suicide Prevention Programs (SPPs) in Israel

Israel has no legislation regarding suicide. An inter-ministerial committee led by the Ministry of Health in the past five years monitors and supports the various coping methods and research is carried out by specific civilian SPPs. This committee initiated a national pilot program, deemed successful at suicide prevention at several sites and with specific populations in Israel. It also established a national council for suicide prevention, which was formed in 2014.

A broad-ranged study examining global literature relating to SPP interventions between the years 1966–2005, found that teaching the gatekeepers, especially primary-care physicians, about early detection and treatment of depression, reduced access to lethal suicide methods and reduced suicide rates [18]. Examples reported in the literature include the reduction of suicide rates in various armies by limiting the availability of lethal weapons [19–21].

Notwithstanding an extensive research literature on suicide risk factors, there are very few publications addressing the effectiveness of SPPs [9–18, 22, 23].

To date, the IDF has accumulated vast knowledge regarding the “suicidal soldier” profile, including: characteristic assignation data, special populations, causes and risk factors unique to the military and to the recruit [11, 24, 25]. Despite rigorous screening, soldiers still commit suicide during their military service for varied reasons. Noteworthy is the fact that only 28 % of military suicides were found to be directly linked to the experience of military service [11].

In 2005, following a relatively high suicide rate in the IDF at that year, a joint venture between the IDF’s mental health department and the Chief of Staff Division developed a suicide-focused prevention program. The IDF SPP began with the commanders being instructed to do whatever possible to lower the suicide rate, by establishing more effective monitoring systems, and by enforcing their implementation.

The IDF Commander-in-Chief’s declaration led to the implementation of two immediate measures. First, a reliable IDF procedure was established for examining suicide incidents through a quality-testing committee, working in synchrony with the routine military police investigations. This new committee’s role was to gather new insights from each suicide event. The second immediate action was the establishment of a civilian advisory team to complement the military one. The civilian advisory team is external to the IDF, and its purpose is to provide a broader perspective, especially a connection to developments in the academic world and to the civilian sector, enabling alternate points of view to those of the military.

### Suicide Prevention Programs (SPPs) in the IDF

From 2006, additional steps were instituted. The IDF’s basic approach to weapon availability was significantly modified, so that accessibility was reduced [8]. Procedures and commands were revised and developed to make commanders more responsible to their soldiers. Another effort focused on providing psycho-educational enrichment for commanders and mental health officers (MHOs) on two axes: *timeline* and *population*. The timeline axis refers to distinct periods during a soldier’s military service career calling for closer monitoring, such as during basic training, the first six months of service, the final six months, prior to discharge, etc. The population axis refers to targeting two specific groups: soldiers characterized by high-risk indicators, such as immigrants and minorities; and training commanders who work with many soldiers for a relatively short durations during sensitive periods for those soldier’s (e.g., during basic training and the initial training of the soldiers’ military occupation). Additional psychoeducational measures included teaching all soldiers the importance of mutual responsibility. In order to enhance the availability of MHO resources to treat the soldiers in need and to instruct the commanders, MHOs were added to the force and were integrated into the military base level as internal MHO operatives, with the goal of reducing the help-seeking stigma among soldiers, their friends, and enabling these MHO operatives to consult the soldiers’ commander as a fellow officer.

### Key components in the evolution of the IDFs SPP

#### Weapon availability reduction policy

In 2006, following the IDF’s Commander-in-Chief’s new directive, the policy was changed, and soldiers were ordered to keep their personal weapons locked in storage when on leave [8].

#### Commands and procedures

Following conclusions made by the quality-testing committee from 2005, several guidelines were instituted and others were revised, based on experience gained by treating soldiers, including soldiers at risk. These orders, formulated by the Mental Health Center, incorporated clinical and army understandings, and were further used as guidelines in the daily routine of MHOs. Among other procedures, these orders dealt with referrals to MHOs, and transfer of medical records between therapists, commanders and families. In addition, the guidelines addressed issues of mental health personnel initiating assessments, treating soldiers in emotional distress, carrying out psychiatric examination recommendations, indicating where service adjustment difficulties exist, carrying out specialist medical recommendations, etc. All these contributed to the appropriate care and protection of the soldier. It should also be noted that in a parallel project, in 2005, all military health care personnel, including the MHOs, began working with a computerized system, so that any IDF medical clinic, no matter where situated, could access the medical history of the soldier, including access to medical examination results and decisions.

#### Timeline axis

The first few stages of military service, such as basic training and army occupation training periods, are regarded as critical adaptation periods. These new transitional situations require coping and adjustment resources, irrespective of the soldier’s perception of it as positive or negative. During this time, the new recruit faces simultaneously a number of lifestyle changes, such as those related to living conditions (transition from home to base); separation from familiar supports (family and friends); and different sleeping and eating arrangements, sometimes with a deficiency in these basic needs. As a result, a high percentage of military suicide cases (38 %) occur within the first six months of military service, particularly during the basic and initial training periods [11, 24]. The data collected from the quality testing committee in relation to the number of suicide cases which occurred soon around the time of discharge from military service, has brought to our attention the fact that the initial service period is not the only critical period, and that the time frame just before discharge from military service is also characterized by an enhanced suicide risk. As a result, adjustments were made, and an identification and detection clause, including coping strategies, was added, with an emphasis on the last year of service as the relevant pre-discharge period for both males and females.

#### Psycho-education

MHOs are the key personnel who are professionally qualified and trained to identify, treat, and prevent suicides within the army. They work both with the individual soldier and with training commanders, helping the latter to detect and identify signs of distress among their troops and intervene immediately.

MHOs are exposed to cumulative and updated knowledge, which they acquire on various training levels, in order to enhance their expertise in conducting clinical assessments of suicide risk. The MHOs are trained to identify and assess risk factors and clinical symptoms, to contact psychiatric hospitals when necessary, or to deal with morbidity within the unit, depending on the risk level and IDF mental health policies.

#### Unit-based MHO

The SPP enabled a new perspective on the role of MHOs and their placement within the military units. The understanding is that there is a need for a MHO as an integrative component of the unit, as an officer familiar with the unit’s servicemen, its commanding staff, and its unique characteristics. The MHO’s effectiveness derives from the understanding that the accessibility of professional personnel increases the awareness among soldiers, facilitates consultation, and reduces the stigma in seeking their help.

#### The population axis

A critical element of the SPP is applying the prevention effort where it would be the most effective. The two main population targets were, therefore, soldier with profiles found in previous studies to be those with the highest suicide rates, and the commanding staff at training bases, thereby enhancing the accessibility for most of the at-risk soldiers during the most sensitive periods. Efforts were made to design psycho-educational programs for these populations as well as for other military personnel in order for them to function as *gate-keepers* [18]. The mental health department established a program for this psycho-educational curriculum, named *“There is a Way”*, which is still functional within the IDF today, eight years after its introduction.

The program aims to train soldiers and especially commanders of all ranks in various training junctures, to detect and identify symptoms of mental illness or overwhelming stress, as well as guiding how to deal with them. It includes training of how to conduct personal interviews and how to use gathering techniques of a wide range of information. These techniques were developed for commanders, with the aim of establishing dialogue channels and interactions with the soldiers under their command. For example, commanders are taught to ask their soldiers direct questions, such as: “Are you feeling depressed?,” “Do you feel that things will not get better?, ” leading up to other direct questions, such as: “Do you want to die?,” “Have you thought of killing yourself?,” etc.

In addition, the IDF Education Corps developed and directs the *mutual responsibility* program, based on the concept of the importance of choosing life and on mutual responsibility. This program was designed to teach commanders to reduce the stigma related to help-seeking from professionals when in distress, and to educate soldiers about mutual responsibility for their own lives as well as for the lives of their peers. In this program, with some overlap, MHOs train commanders to raise their awareness and to teach soldiers to recognize, in both themselves and in their peers, distress and other behaviors that may signal a suicidal intent. The overall message is to look after themselves and their peers, and, if they do identify signs of distress requiring an intervention of a commander or a MHO they should not hesitate and seek help. The peer group is essential today more than ever, since often, distress appears in venues such as *social media* and not in letters or other traditional forms of expression. These signs would be more conspicuous to friends than to commanders or caregivers. Both programs emphasize the preparedness of training base staff as avenues of hope and change.

## Conclusions

In this paper we extensively reviewed the IDF Suicide Prevention Program, established in 2005. The program has since been refined and expanded, currently encompassing additional professional fields such as categorization and assignation. The SPP is based on the above mentioned procedures and was designed in a modular fashion, enabling various components within it to continue and evolve. The SPP is backed by the military policy that supports its implementation, starting with the commanders being guided to do whatever possible to lower the suicide rate, building deeper and faster monitoring systems and enforcing their implementation. The IDF Suicide Prevention Program brought to the reduction of suicide rates by almost 50 % from 2006 to 2014. This decline is also reflected in the Israeli National Statistics Registry for the same period of time among 18–21 year-olds [26].

In a statistical examination of the SPP, trend analysis showed lower suicide rates in the cohort subsequent to intervention (Shelef L, Laur L, Derazne E, Mann JJ, Fruchter E. An effective suicide prevention program in the Israel Defense Force: a cohort study. In preparation). The hazard ratio for the SPP’s effect on time-to-suicide was 0.44 (95 % CI =0.34–0.56, *p* < .001). The IDF SPP reduced the suicide rate by 43 %. Several other variables were found to mediate the effect of the SPP: male gender, place of birth, high socioeconomic status, high intellectual level, and service in a combat unit (Shelef L, Laur L, Derazne E, Mann JJ, Fruchter E. An effective suicide prevention program in the Israel Defense Force: a cohort study. In preparation). Future efforts should seek to extend the program’s prevention reach to other demographic soldier profiles. The success of the IDF program may inform other suicide prevention programs in other military organizations and in the civilian sector.
